# Stegomyia indices and pattern recognition of *Aedes aegypti* (*Diptera*: *Culicidae*) in selected agrogeoclimatic zones of Punjab, Pakistan

**DOI:** 10.1016/j.sjbs.2023.103919

**Published:** 2023-12-23

**Authors:** Muhammad Abdullah Malik, Muhammad Sohail Sajid, Rasha Khalifah Al-Akeel, Mughees Aizaz Alvi, Hafiz Muhammad Rizwan, Haider Abbas, Mahvish Maqbool

**Affiliations:** a Department of Parasitology, Faculty of Veterinary Science, University of Agriculture, Faisalabad, Pakistan; b Department of Zoology, King Saud University, Riyadh, Saudi Arabia; c Department of Clinical Medicine and Surgery, Faculty of Veterinary Science, University of Agriculture, Faisalabad, Pakistan; d Section of Parasitology, Department of Pathobiology, KBCMA College of Veterinary and Animal Sciences, Narowal, Sub Campus UVAS, Lahore, Pakistan

**Keywords:** *Aedes aegypti*, Breeding habitats, Ecology, *Stegomyia* indices, Water containers

## Abstract

Mosquito-borne diseases especially, dengue is gaining currency nowadays in Pakistan. As there is no approved dengue vaccine available worldwide, prevention and control of vector is the only solution amid prevailing circumstances. The present study is a maiden attempt to screen indoor and outdoor breeding containers for the presence of Aedes (*Ae.*) aegypti larvae from selected study districts of Punjab, Pakistan i.e., Dera Ghazi Khan (DG Khan), Chakwal, and Faisalabad. A total of 384 houses from each study districts were surveyed for a calendar year. Mosquito larvae were collected, preserved, and identified using standard taxonomic keys. House Index (HI), Container Index (CI), and Breteau Index (BI) were estimated. Chi-square analysis was applied to calculate the association between *Ae. aegypti* larvae and breeding containers. Chakwal was identified with the highest values of Stegomyia indices (HI = 46.61 %, BI = 91.67 %, and CI = 15.28 %) followed by Faisalabad (HI = 34.11 %, BI = 68.75 % and, CI = 13.04 %) and DG Khan (HI = 28.39 %, BI = 68.23 % and, CI = 11.29 %). Earthen jars, tree holes, and water tanks were found significantly (p < 0.05) associated with the abundance of larvae irrespective of the geographical location. However, flower tubs and plastic buckets were found significantly (p < 0.05) associated in Faisalabad and Chakwal while, tyres and plastic bottles were found associated (p < 0.05) with the abundance of *Ae. aegypti* larvae in Faisalabad and DG Khan. These findings will help the stakeholders to devise appropriate preventive measures in combating the risk of dengue transmission.

## 1 Introduction

Mosquitoes, especially *Ae*. *aegypti* (Linnaeus) is distributed worldwide. It usually breeds in indoor and outdoor breeding sites either made naturally or artificially e.g., water tanks, tree holes, tyres, earthen jars, clay pots, flower vases, plastic sheets, plastic bottles, plant axils etc. In outdoor settings, breeding sites are created out of neglected areas of construction, and stagnant water either of rain or tap water which create favorable conditions for breeding of mosquitoes (Elsinga et al., 2018).

The risk of mosquito-borne disease transmission is influenced by multiple factors i.e., mobility of goods, human migration, climatic changes, population density, deforestation, urbanization, presence of invasive vectors and pathogens, vectoral capacity, and insufficient control strategies (Manikandan et al., 2023). As an approved vaccine against dengue has not been launched yet in the country, vector surveillance and control remain the only ways to contain dengue transmission at mass level (Nakase et al., 2023). To provide a measurable quantity of dengue virus transmission and spatial distribution, WHO recommends a routine vector surveillance to determine the risk of outbreaks and probable interventions (WHO, 2009). In these quantifiable measures, *Stegomyia* indices i.e., HI, CI, and BI are used to predict disease outbreaks likely caused by mosquito *Ae*. *aegypti* (Borah and Bora, 2022). These indices are estimates of number of mosquito larvae present or absent in potential breeding containers either in indoor or outdoor settings in and around each house. The calculation of these indices is not only helpful in identifying the areas at high risk of *Aedes*-borne diseases, but also help to formulate appropriate control measures and their implementation in areas at risk. These measures may include release of larvae predators in water holding containers, elimination of breeding habitats and breeding containers from indoor and outdoor settings, and spray of insecticides.

Therefore, to effectively control the population of mosquitoes and harness the mosquito-borne diseases, it is necessary to increase the knowledge of potential breeding habitats of *Aedes* mosquitoes (Souza et al., 2019). In Pakistan, despite of increase in surveillance of mosquitoes and mosquito-borne diseases, the association of potential breeding containers in indoor and outdoor settings with the abundance of *Ae. aegypti* larvae has not been established in the selected study areas so far. Therefore, the present investigation is a maiden attempt in establishing the association of potential breeding containers with the abundance of *Ae. aegypti* larvae and investigation of *Stegomyia* indices of the selected areas. The evidence generated here will be vital for undertaking early prevention and control interventions.

## 2 Materials and methods

### 2.1 Study area and survey sites

**Fig. 1:**
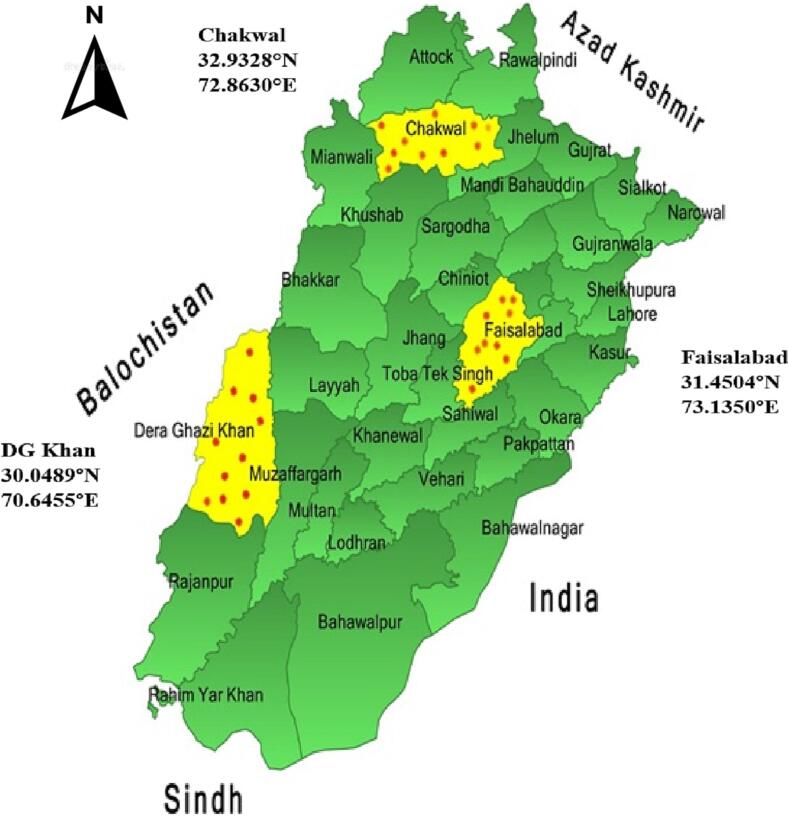
Physical map of Punjab showing sampling sites (red spots) in Chakwal, Faisalabad and DG Khan Districts of Punjab, Pakistan.

In this proposal, three districts of Punjab (Chakwal, Faisalabad, and Dera Ghazi Khan) representative of the three different agro geo-climatic zones (North, Central and South respectively) of Punjab province were selected for sampling of *Ae. aegypti* larvae (Fig. 1). A pilot survey was conducted in the selected study districts to identify the potential breeding sites of the *Ae. aegypti* (Thrusfield et al., 2018).

### 2.2 Development of questionnaire

The questionnaire was developed and refined through formal and informal testing. Intra-observer variation was dealt through variability measurement through standard deviation. The data obtained was shifted to excel data sheets and statistical tools were applied to investigate any association between the prevalence of *Ae. aegypti* larvae and associated risk factors (Thrusfield et al., 2018).

### 2.3 Classification of water containers

Based on pilot survey, all containers were divided into two major categories (i) indoor containers (located inside the houses screened) and (ii) outdoor containers (located outside of the houses or in the field area near by human or animal population. The list of containers is mentioned in Table 1:

**Table 1:** Classification of breeding containers based on their locations i.e., indoor/outdoor.

Sr. No.	Indoor Containers	Outdoor Containers
	Refrigerator tray	Tyres
	Earthen Jar	Water tank
	Plastic drum	Plastic bottles
	Flower tubs	Tree holes
	Plastic bucket	Plastic sheets
	Clay pot	Plastic bags
		Plant axil

### 2.4 Examination of the water containers

All containers inside the houses as well as outside area were investigated for the presence of *Ae. aegypti* larvae. The respondents were asked about the source and use of water within each container. Any outdoor container having water without nearby water source was considered rainwater. *Stegomyia* indices including House Index (HI), Breteau Index (BI), Container Index (CI), and their 95 % confidence interval were calculated. The following formulae were used:

$$HI=\frac{Number\;of\;houses\;infested}{Total\;number\;of\;houses\;inspected}\times 100$$

$$CI=\;\frac{Number\;of\;positive\;containers}{Total\;number\;of\;containers\;inspected}\times 100$$

$$BI=\frac{Number\;of\;positive\;containers}{Total\;number\;of\;households\;inspected}\times 100$$

The areas were considered at high risk of mosquito-borne disease transmission, if the threshold of HI and BI found more than 5 % and of CI found more than 3 %.

### 2.5 Collection of mosquitoes

A total of 384 houses from each study districts were surveyed for a calendar year 2018–19. A systematic random sampling technique was used. First house was randomly selected for screening. Thereafter, every 10th house was screened for the presence of potential breeding habitats (Thrusfield et al., 2018). All the contents of the containers were shifted to large trays. The immatures were picked through plastic cup. The larvae collected were transferred to the laboratory for rearing purpose.

### 2.6 Rearing and taxonomic identification of *Aedes aegypti*

Mosquito larvae were raised at temperature of 27 °C with relative humidity of 80 % and a day/night period of 12 h. The adults emerged from pupae were identified based on arrangement of white stripes using identification guides under stereomicroscope (Harrison, 2005, Rueda, 2007).

### 2.7 Statistical analysis

The data obtained from survey was statistically analyzed through SPSS statistical software package version 25.0 (Corp, 2017). The information collected from field survey was added to the questionnaire and shifted to the excel data sheets. The population of *Ae. aegypti* was considered as a dependent variable while, different types of breeding containers (plastic bottles, bags, sheets, drums, buckets, flower tubs, refrigerator trays, tree holes, plant axils, earthen jar, water tanks, clay pots and tyres) were considered as an independent variable. Chi-square test was applied to calculate the Odds Ratio (OR). The value of OR > 1 was considered significant and showed the sampling area is at high risk while OR < 1 was considered non-significant and showed no significant association between the breeding containers and population of *Aedes* immatures. The probability value (P-value) of less than 0.05 % was considered effective to accept or reject the hypothesis with a 95 % Confidence Interval.

## 3 Results

The presence of water holding containers indoor and outdoor at the selected study sites permits larvae of *Ae. aegypti* to breed which ultimately results in increased population of *Aedes* and transmission of arbovirus. In all the selected study districts, the *Stegomyia* indices HI, BI and CI were higher than the threshold level indicated by WHO (Anonymous, 2009). The HI, BI and CI of district Faisalabad were 34.11 %, 68.75 % and 13.04 % respectively. Similarly, HI, BI and CI for district DG Khan were 28.39 %, 68.23 % and 11.29 % respectively. In Chakwal, these larval indices were recorded highest among all the study districts i.e., 46.61 %, 91.67 % and 15.28 % respectively. The Stegomyia indices mapped through GIS are shown in Fig. 9.

### 3.1 Plastic drums

**Fig. 2:**
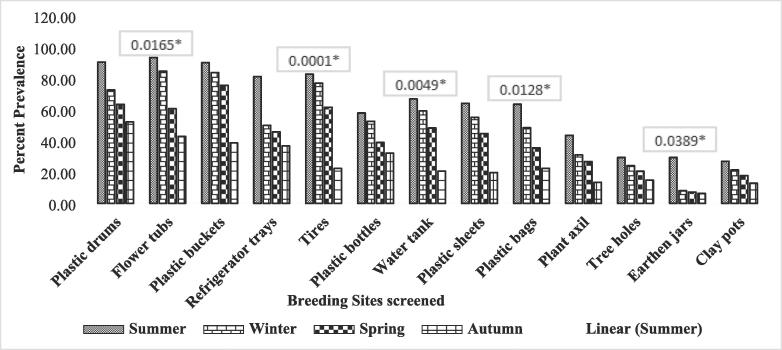
Association of different factors with prevalence of Aedes aegypti in Faisalabad isolated during different seasons.

**Fig. 3:**
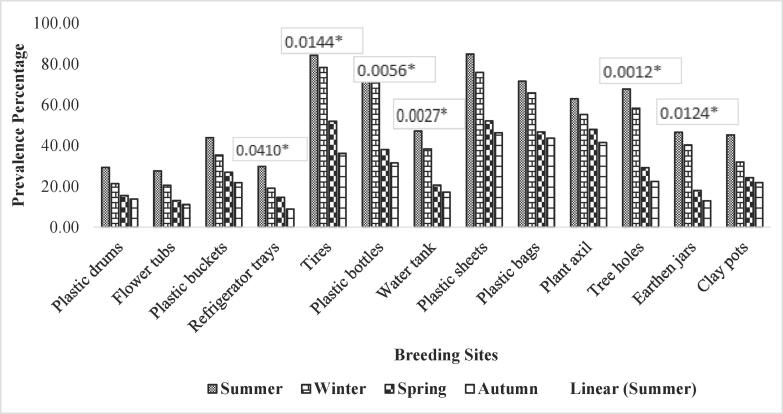
Association of different factors with prevalence of Aedes aegypti in DG Khan isolated during different seasons.

**Fig. 4:**
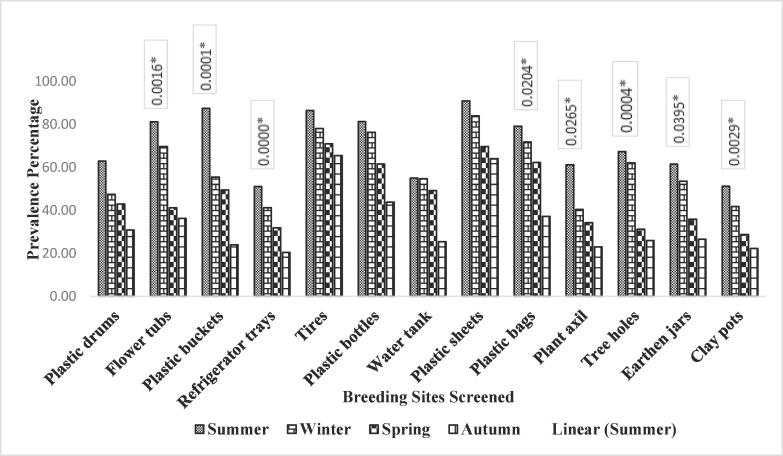
Association of different factors with prevalence of Aedes aegypti in Chakwal isolated during different seasons.

In Faisalabad district, the association of plastic drums with the abundance of *Ae*. *aegypti* larvae throughout the year was found statistically insignificant with Odds Ratio (OR) = 1.73, P-value = 0.6425 as described in Fig. 2 and Table 2. Similarly, plastic drums screened for the presence of *Ae. aegypti* larvae in district Dera Ghazi Khan have shown insignificant association with the abundance of mosquitoes (OR = 4.54, P-value = 0.2084) as shown in Fig. 3 and Table 3. On the contrary, plastic drums screened for the presence of larvae in district Chakwal have been found significantly associated with the production of larvae of the mosquito (OR = 2.10, P-value = 0.5515) shown in table and Fig. 4.Table 4.

**Table 2:** Association of different factors with prevalence of *Ae. aegypti* in Faisalabad isolated during different seasons in the year 2018–19.

Factor	Levels	Positive	Total	Prevalence (%)	OR	95 % CI	χ^2^	p-value
Plastic drums	Summer	19	21	90.47619	1.73	0.69–4.36	1.67	0.6425
	Spring	61	84	72.61905	1.25	0.62–2.50		
	Winter	52	82	63.41463	1.43	0.71–2.88		
	Autumn	12	23	52.17391	–	–		
Flower tubs	Spring	113	121	93.38843	2.18	0.97–4.89	10.26	0.0165*
	Summer	11	13	84.61538	1.10	0.48–2.52		
	Winter	37	61	60.65574	1.54	0.95–2.49		
	Autumn	9	21	42.85714	–	–		
Plastic buckets	Spring	73	81	90.12346	2.32	1.26–4.29	7.69	0.0529
	Summer	31	37	83.78378	1.08	0.61–1.90		
	Winter	59	78	75.64103	1.19	0.75–1.89		
	Autumn	19	49	38.77551	–	–		
Refrigerator trays	Summer	13	16	81.25	2.71	1.10–6.70	3.16	0.3677
	Autumn	9	18	50	2.00	0.67–5.94		
	Spring	27	59	45.76271	2.19	0.91–5.26		
	Winter	21	57	36.84211	–	–		
Tyres	Spring	173	209	82.77512	3.70	2.05–6.68	21.17	0.0001*
	Winter	107	139	76.97842	1.08	0.78–1.48		
	Summer	24	39	61.53846	1.35	0.78–2.32		
	Autumn	15	67	22.38806	–	–		
Plastic bottles	Summer	11	19	57.89474	1.80	0.81–4.00	5.35	0.1480
	Spring	61	116	52.58621	1.10	0.50–2.43		
	Autumn	16	41	39.02439	1.48	0.59–3.75		
	Winter	47	146	32.19178	–	–		
Water tank	Spring	97	145	66.89655	3.23	1.66–6.31	12.90	0.0049*
	Winter	61	103	59.2233	1.13	0.75–1.70		
	Summer	14	29	48.27586	1.39	0.70–2.74		
	Autumn	12	58	20.68966	–	–		
Plastic sheets	Spring	191	298	64.09396	3.25	1.79–5.91	2.51	0.4740
	Winter	59	107	55.14019	1.16	0.81–1.67		
	Summer	17	38	44.73684	1.43	0.79–2.60		
	Autumn	14	71	19.71831	–	–		
Plastic bags	Spring	113	178	63.48315	2.83	1.42–5.64	10.81	0.0128*
	Winter	31	64	48.4375	1.31	0.81–2.13		
	Summer	11	31	35.48387	1.90	0.93–3.89		
	Autumn	11	49	22.44898	–	–		
Plant axil	Winter	17	39	43.58974	3.24	1.23–8.50	5.99	0.1122
	Summer	9	29	31.03448	1.40	0.55–3.56		
	Spring	26	97	26.80412	1.63	0.80–3.31		
	Autumn	7	52	13.46154	–	–		
Tree holes	Summer	5	17	29.41176	1.97	0.67–5.79	3.32	0.3447
	Spring	43	178	24.1573	1.22	0.43–3.41		
	Autumn	7	34	20.58824	1.43	0.40–5.05		
	Winter	21	141	14.89362	–	–		
Earthen jars	Summer	5	17	29.41176	4.63	1.47–14.58	8.38	0.0389*
	Autumn	3	37	8.108108	3.63	0.80–16.52		
	Spring	17	234	7.264957	4.05	1.36–12.05		
	Winter	11	173	6.358382	–	–		
Clay pots	Spring	21	78	26.92308	2.09	0.67–6.48	2.19	0.5338
	Summer	3	14	21.42857	1.26	0.34–4.62		
	Winter	13	73	17.80822	1.51	0.71–3.22		
	Autumn	4	31	12.90323	–	–

**Table 3:** Association of different factors with prevalence of *Ae. aegypti* in Dera Ghazi Khan isolated during different seasons in the year 2018–19.

Factor	Levels	Positive	Total	Prevalence (%)	OR	95 % CI	χ^2^	p-value
Plastic drums	Spring	54	184	29.34783	2.13	0.73–6.22	4.54	0.2084
	Summer	21	98	21.42857	1.37	0.78–2.39		
	Autumn	9	58	15.51724	1.89	0.88–4.04		
	Winter	4	29	13.7931	–	–		
Flower tubs	Spring	61	221	27.60181	2.48	0.74–8.30	3.74	0.2909
	Summer	7	34	20.58824	1.34	0.57–3.14		
	Autumn	3	23	13.04348	2.12	0.63–7.12		
	Winter	3	27	11.11111	–	–		
Plastic buckets	Spring	47	107	43.92523	2.01	0.84–4.83	3.71	0.2951
	Summer	17	48	35.41667	1.24	0.65–2.37		
	Autumn	14	52	26.92308	1.63	0.83–3.21		
	Winter	7	32	21.875	–	–		
Refrigerator trays	Summer	14	47	29.78723	3.33	1.42–7.82	8.26	0.0410*
	Autumn	9	47	19.14894	1.56	0.62–3.91		
	Winter	6	41	14.63415	2.04	0.72–5.72		
	Spring	11	123	8.943089	–	–		
Tyres	Spring	113	134	84.32836	2.33	1.27–4.27	10.56	0.0144*
	Summer	54	69	78.26087	1.08	0.70–1.66		
	Autumn	41	79	51.89873	1.62	1.03–2.55		
	Winter	17	47	36.17021	–	–		
Plastic bottles	Spring	71	91	78.02198	2.47	1.34–4.55	12.60	0.0056*
	Summer	17	24	70.83333	1.10	0.55–2.19		
	Autumn	27	71	38.02817	2.05	1.20–3.52		
	Winter	18	57	31.57895	–	–		
Water tank	Spring	134	284	47.1831	2.75	1.41–5.35	14.17	0.0027*
	Summer	41	107	38.31776	1.23	0.81–1.86		
	Autumn	12	58	20.68966	2.28	1.19–4.37		
	Winter	11	64	17.1875	–	–		
Plastic sheets	Spring	134	158	84.81013	1.83	1.02–3.29	7.15	0.0674
	Summer	47	62	75.80645	1.12	0.72–1.74		
	Autumn	37	71	52.11268	1.63	1.03–2.57		
	Winter	19	41	46.34146	–			
Plastic bags	Spring	91	127	71.65354	1.64	1.00–2.70	5.24	0.1549
	Summer	27	41	65.85366	1.09	0.63–1.89		
	Autumn	29	62	46.77419	1.53	0.92–2.56		
	Winter	31	71	43.66197	–	–		
Plant axil	Autumn	17	27	62.96296	1.52	0.79–2.92	2.10	0.5515
	Summer	21	38	55.26316	1.14	0.51–2.53		
	Winter	23	48	47.91667	1.31	0.60–2.86		
	Spring	78	188	41.48936	–	–		
Tree holes	Autumn	21	31	67.74194	3.01	1.60–5.67	15.83	0.0012*
	Summer	14	24	58.33333	1.16	0.50–2.72		
	Winter	21	72	29.16667	2.32	1.12–4.83		
	Spring	47	209	22.48804	–	–		
Earthen jars	Spring	41	88	46.59091	3.61	1.21–10.75	10.88	0.0124*
	Summer	27	67	40.29851	1.16	0.65–2.06		
	Autumn	11	61	18.03279	2.58	1.24–5.40		
	Winter	4	31	12.90323	–	–		
Clay pots	Spring	91	201	45.27363	2.06	0.97 to 4.39	6.25	0.0999
	Summer	31	97	31.95876	1.42	0.88–2.27		
	Autumn	8	33	24.24242	1.87	0.84–4.16		
	Winter	9	41	21.95122	–	–

**Table 4:** Association of different factors with prevalence of *Ae. aegypti* in Chakwal isolated during different seasons in year 2018–19.

Factor	Levels	Positive	Total	Prevalence (%)	OR	95 % CI	χ^2^	p-value
Plastic drums	Spring	78	124	62.90323	2.04	1.20–3.49	7.72	0.0522
	Summer	91	192	47.39583	1.33	0.91–1.93		
	Autumn	54	126	42.85714	1.47	0.96–2.25		
	Winter	24	78	30.76923	–	–		
Flower tubs	Spring	112	138	81.15942	2.24	1.29–3.90	15.33	0.0016*
	Summer	78	112	69.64286	1.17	0.80–1.71		
	Autumn	46	112	41.07143	1.98	1.29–3.02		
	Winter	21	58	36.2069	–	–		
Plastic buckets	Summer	90	103	87.37864	3.65	2.01–6.63	21.23	0.0001*
	Autumn	41	74	55.40541	1.58	0.98–2.53		
	Spring	87	176	49.43182	1.77	1.21–2.59		
	Winter	17	71	23.94366	–	–		
Refrigerator trays	Spring	48	94	51.06383	2.51	1.21–5.21	28.91	0.0000*
	Summer	44	107	41.1215	1.24	0.76–2.03		
	Autumn	27	85	31.76471	1.61	0.92–2.79		
	Winter	11	54	20.37037	–	–		
Tyres	Spring	178	206	86.40777	1.32	0.88–1.98	2.55	0.4665
	Summer	131	168	77.97619	1.11	0.82–1.50		
	Autumn	112	158	70.88608	1.22	0.89–1.67		
	Winter	51	78	65.38462	–	–		
Plastic bottles	Spring	91	112	81.25	1.86	1.04–3.31	5.16	0.1605
	Summer	64	84	76.19048	1.07	0.70–1.63		
	Autumn	48	78	61.53846	1.32	0.84–2.08		
	Winter	21	48	43.75	–	–		
Water tank	Summer	112	204	54.90196	2.16	1.22–3.85	7.79	0.0505
	Spring	141	258	54.65116	1.00	0.74–1.37		
	Autumn	89	181	49.17127	1.12	0.79–1.57		
	Winter	17	67	25.37313	–	–		
Plastic sheets	Spring	178	196	90.81633	1.42	0.91–2.22	4.24	0.2363
	Summer	141	168	83.92857	1.08	0.80–1.46		
	Autumn	119	171	69.59064	1.31	0.96–1.78		
	Winter	39	61	63.93443	–	–		
Plastic bags	Spring	117	148	79.05405	2.13	1.30–3.47	9.79	0.0204*
	Summer	89	124	71.77419	1.10	0.77–1.58		
	Autumn	66	106	62.26415	1.27	0.86–1.88		
	Winter	29	78	37.17949	–	–		
Plant axil	Summer	41	67	61.19403	2.67	1.33–5.34	9.22	0.0265*
	Autumn	23	57	40.35088	1.52	0.82–2.81		
	Spring	51	149	34.22819	1.79	1.08–2.95		
	Winter	14	61	22.95082	–	–		
Tree holes	Autumn	39	58	67.24138	2.59	1.28–5.27	17.98	0.0004*
	Summer	54	87	62.06897	1.08	0.64–1.83		
	Spring	71	228	31.14035	2.16	1.33–3.50		
	Winter	14	54	25.92593	–	–		
Earthen jars	Spring	91	148	61.48649	2.32	1.20–4.48	8.34	0.0395*
	Summer	31	58	53.44828	1.15	0.69–1.91		
	Autumn	19	53	35.84906	1.72	0.96–3.07		
	Winter	13	49	26.53061	–	–		
Clay pots	Spring	103	201	51.24378	2.31	1.32–4.04	13.97	0.0029*
	Summer	73	175	41.71429	1.23	0.86–1.76		
	Autumn	51	178	28.65169	1.79	1.21–2.64		
	Winter	18	81	22.22222	–	–		

### 3.2 Flower tubs

Statistically significant association has been found among larval population of *Ae. aegypti* and flower tubs screened throughout the year in district Faisalabad with OR = 2.18, P-value = 0.0165 as shown in table and Fig. 2. Similarly, significant association has been found between prevalence of *Ae. aegypti* larvae and flower tubs screened in district Chakwal (OR = 15.33, P-value = 0.0016) shown in table and Fig. 4. However, insignificant association has been found between flower tubs screened in Dera Ghazi Khan for the prevalence of *Ae. aegypti* larvae (OR = 3.74, P-value = 0.2909) as shown in table and Fig. 3.

### 3.3 Plastic buckets

Statistically significant association has been found between abundance of *Ae. aegypti* larvae in plastic bucket containers in both districts Faisalabad and Chakwal with OR = 7.69, P-value = 0.0529. and OR = 21.23, P-value = 0.0001, respectively shown in table and Fig. 2, Fig. 4, respectively. However, insignificant association has been found with plastic buckets screened for the presence of *Ae. aegypti* larvae in district Dera Ghazi Khan with OR = 3.71 and P-value = 0.2951 as shown in table and Fig. 3.

### 3.4 Refrigerator trays

The refrigerator trays screened during the year 2018–19 in district Faisalabad were found statistically non-associated with abundance of *Ae. aegypti* larvae with OR = 2.71, P-value = 0.3677 mentioned in table and Fig. 2. However, significant association was found between refrigerator trays screened for the prevalence of *Ae. aegypti* larvae in DG Khan and Chakwal districts with OR = 8.26, P-value = 0.0410 and OR = 15.33, P-value = 0.0016, respectively shown in table and Fig. 3, Fig. 4.

### 3.5 Tyres

Irrespective of seasonal variation, a significant statistical association has been found between the prevalence of *Ae. aegypti* larvae and tyres screened from collection sites in district Faisalabad with OR = 3.70, P-value = 0.0001 as shown in table and Fig. 2. Similarly, significant association has been found between tyres screened in district DG Khan and prevalence of *Ae. aegypti* larvae shown in table and Fig. 3 (OR = 10.56, P-value = 0.0144). However, no significant association has been observed between tyres screened in district Chakwal and abundance of larvae of the *Ae. aegypti* shown in table and Fig. 4 (OR = 2.56, P-value = 0.4665).

### 3.6 Plastic bottles

The data obtained after screening of plastic bottles from selected collection sites was statistically analyzed and no significant association found among the population of *Ae. aegypti* larvae and plastic bottles screened throughout the year in district Faisalabad (OR = 1.80, P-value = 0.1480) shown in table and Fig. 2. Similarly, in district Chakwal, no significant association has been observed between the larvae of *Ae. aegypti* and plastic bottles screened in the study year (OR = 5.16, P-value = 1.605) described in table and Fig. 4. However, significant association was found among the plastic bottles screened and *Ae*. *aegypti* larvae collected from district DG Khan (OR = 12.60, P-value = 0.056) as shown in table and Fig. 3.

### 3.7 Water tanks

Water tanks screened from collection sites in Faisalabad district were found statistically associated with the population of *Ae. aegypti* larvae throughout the year 2018–19 (OR = 3.23, P-value = 0.0049) as shown in table and Fig. 2. Similarly, significant association was found among *Ae. aegypti* larvae and the water tanks screened at selected sites in district DG Khan (OR = 14.17, P-value = 0.0027) and Chakwal (OR = 7.79, P-value = 0.0505) as shown in table and Fig. 3, Fig. 4, respectively.

### 3.8 Plastic sheets

The plastic sheets that are used to cover hay stocks, silage, AC outdoors and other consumables were screened for the presence of *Ae. aegypti* larvae. Irrespective of the geographical locations, the data analyzed, showed no significant statistical association between the abundance of the mosquito and plastic sheets screened throughout the year in all the selected study districts i.e., Faisalabad (OR = 3.25, P-value = 0.4740), DG Khan (OR = 7.15, P-value = 0.0674), and Chakwal (OR = 4.24, P-value = 0.2363) given in table and Fig. 2, Fig. 3, Fig. 4, respectively.

### 3.9 Plastic bags

Plastic bags that are thrown aimlessly without proper disposal in garbage, placed on the corners of the floor or roofs of the rooms were screened alongside plastic sheets. The analyses showed significant association between *Ae. aegypti* larvae and plastic bags screened from collection sites throughout the year in district Faisalabad (OR = 2.83, P-value = 0.0128) shown in table and Fig. 2. Similarly, in district Chakwal significant association has been observed between *Ae. aegypti* larvae and plastic bags screened throughout the study period (OR = 9.79, P-value = 0.0204) given in table and Fig. 4. However, no significant association was found between plastic bags and population of *Ae. aegypti* larvae in district DG Khan (OR = 5.24, P-value = 0.1549) as shown in table and Fig. 3.

### 3.10 Plant axil

In the field area, mosquito preferred breeding sites were also screened like plant axil. The collected mosquito population was screened for the quantification of *Ae aegypti* larvae and its association with the breeding site. The statistical analyses showed non-significant association between *Ae. aegypti* larvae and plant axil screened throughout the year in district Faisalabad (OR = 3.24, P-value = 0.1122) shown in table and Fig. 2. Similarly, no significant association was observed between *Ae. aegypti* larvae and plant axil screened in district DG Khan (OR = 2.10, P-value = 0.5515) given in table and Fig. 3. However, significant association was observed between plant axil and abundance of *Ae. aegypti* larvae while screening in district Chakwal (OR = 9.22, P-value = 0.0265) as shown in table and Fig. 4.

### 3.11 Tree holes

Tree holes could be another preferred site for *Ae. aegypti* mosquito for breeding. To assess the association between *Ae. aegypti* larvae and tree holes, the breeding material was screened for the presence of mosquito larvae. However, no statistical association has been found between *Ae. aegypti* larvae and tree holes screened for the year in district Faisalabad (OR = 1.97, P-value = 0.3447) given in table and Fig. 2. However, significant association has been observed among the tree holes and prevalence of *Ae. aegypti* larvae isolated from both districts DG Khan (OR = 15.83, P-value = 0.0012) and Chakwal (OR = 17.98, P-value = 0.0004) as given in table and Fig. 3, Fig. 4, respectively.

### 3.12 Earthen jars

Earthenware is usually utilized to store fresh drinking water and kitchen gardening etc. These are also preferred breeding sites for the *Ae. aegypti* due to availability of shade, moisture, humidity, and ambient temperature. The earthen jars were screened for the presence of *Ae. aegypti* larvae throughout the year in district Faisalabad. The data obtained was statistically analyzed which reveals significant association in all the selected study districts i.e., Faisalabad (OR = 4.63, P-value = 0.0389), DG Khan (OR = 10.88, P-value = 0.0124), and Chakwal (OR = 8.34, P-value = 0.0395) as shown in table and Fig. 2, Fig. 3, Fig. 4, respectively.

### 3.13 Clay pots

Clay pots is another earthenware which is preferred by *Ae. aegypti* for its breeding. The clay pots placed on the roofs with water inside them for the facilitation of birds in extreme summer specifically and throughout year usually also provide a chance to breed and increase the population. However, clay pots screened during the year 2018–19 in district Faisalabad showed no statistical association between the prevalence of *Ae. aegypti* larvae (OR = 2.09, P-value = 0.5338) given in table and Fig. 2. Similarly, no significant association has been observed between clay pots and *Ae. aegypti* larvae investigated in district DG Khan throughout the study period (OR = 6.25, P-value = 0.0999) shown in table and Fig. 3. However, significant association was observed between clay pots of Chakwal screened for the abundance of *Ae. aegypti* larvae throughout study period (OR = 13.97, P-value = 0.0029) as shown in table and Fig. 4.

It has been reported that consistent population of the *Ae. aegypti* larvae was present in the supply lines or inside the sealed containers throughout winter season (Fig. 5a. and Fig. 5b.) when the conditions were not favorable which shows that roof piping might have played the role in seeding water tanks with mosquito larvae during favorable conditions i.e., rainfall etc. (Staunton et al., 2020). The association of other seasons (Summer, Autumn and Spring) has also been shown in Figs. 6a, 6b., 7a., 7b., 8a., and 8b. below.

**Fig. 5a:**
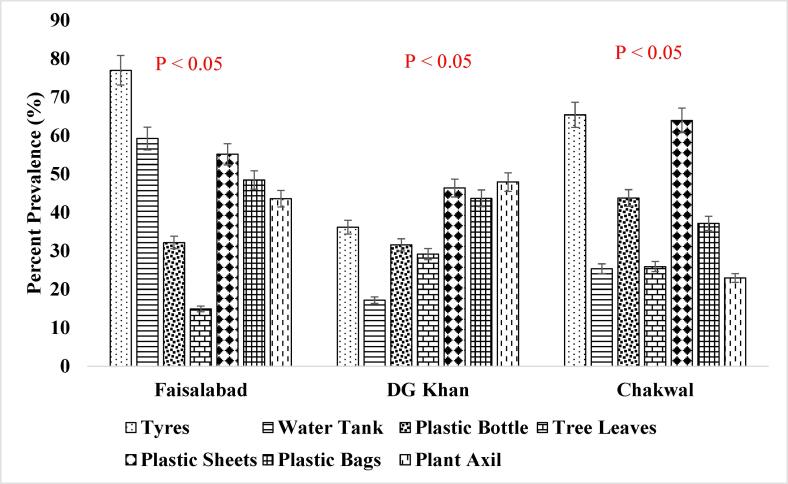
Percent association of winter season with the outdoor breeding containers for the presence of aedes aegypti in selected agrogeoclimatic zones of punjab, Pakistan.

**Fig. 5b:**
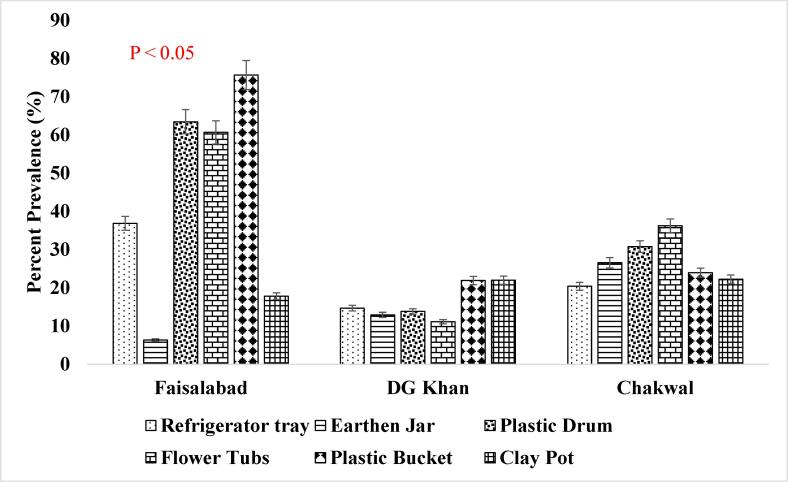
Percent association of winter season with the indoor breeding containers for the presence of Aedes aegypti in selected agrogeoclimatic zones of Punjab, Pakistan.

**Fig. 6a:**
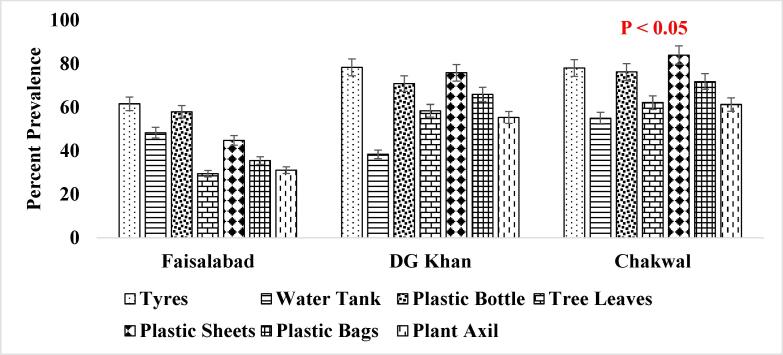
Percent association of summer season with the outdoor breeding containers for the presence of *Aedes aegypti* in selected agrogeoclimatic zones of Punjab, Pakistan.

**Fig. 6b:**
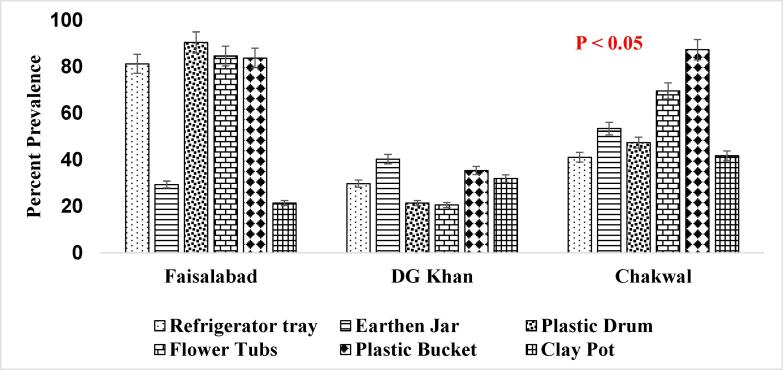
Percent association of summer season with the indoor breeding containers for the presence of *Aedes aegypti* in selected agrogeoclimatic zones of Punjab, Pakistan.

**Fig. 7a:**
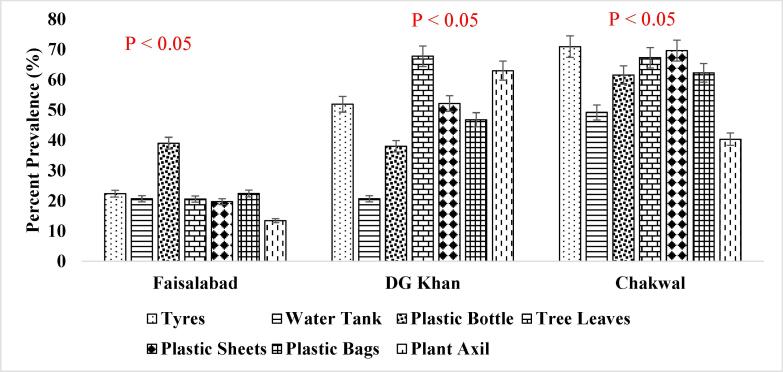
Percent association of autumn season with the outdoor breeding containers for the presence of aedes aegypti in selected agrogeoclimatic zones of punjab, Pakistan.

**Fig. 7b:**
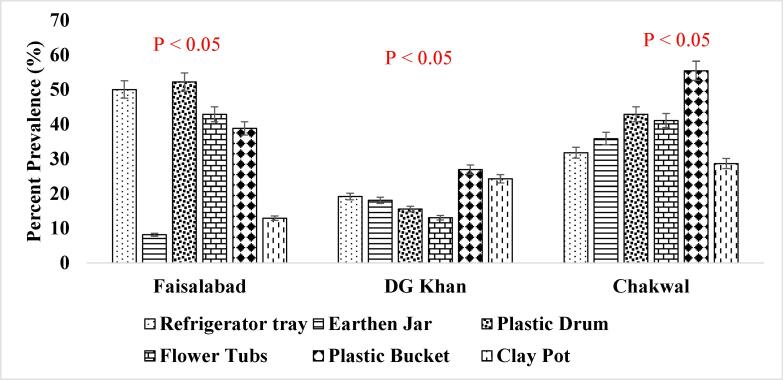
Percent association of autumn season with the indoor breeding containers for the presence of aedes aegypti in selected agrogeoclimatic zones of punjab, pakistan.

**Fig. 8a:**
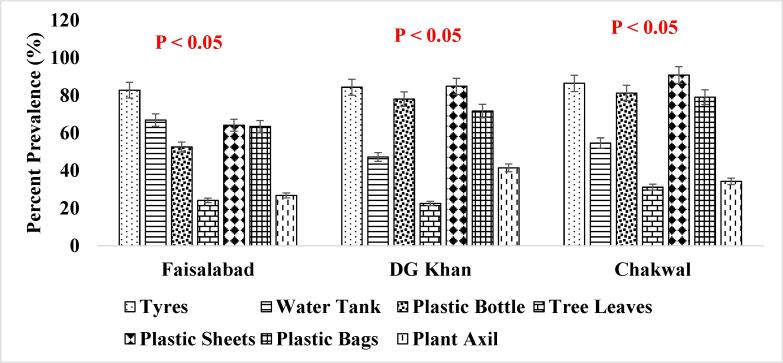
Percent association of spring season with the outdoor breeding containers for the presence of aedes aegypti in selected agrogeoclimatic zones of punjab, Pakistan.

**Fig. 8b:**
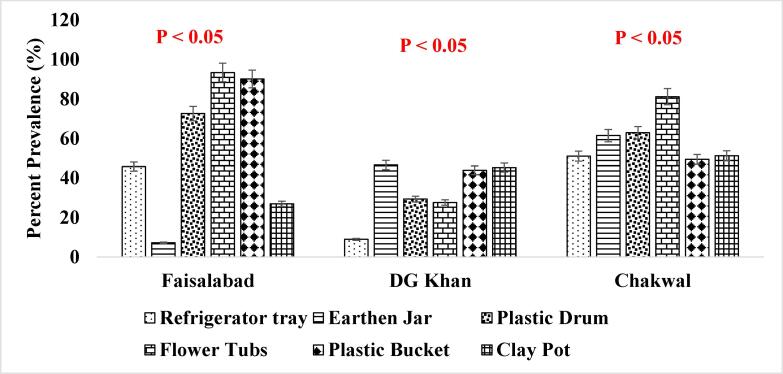
Percent association of spring season with the indoor breeding containers for the presence of Aedes aegypti in selected agrogeoclimatic zones of Punjab, Pakistan.

**Fig. 9:**
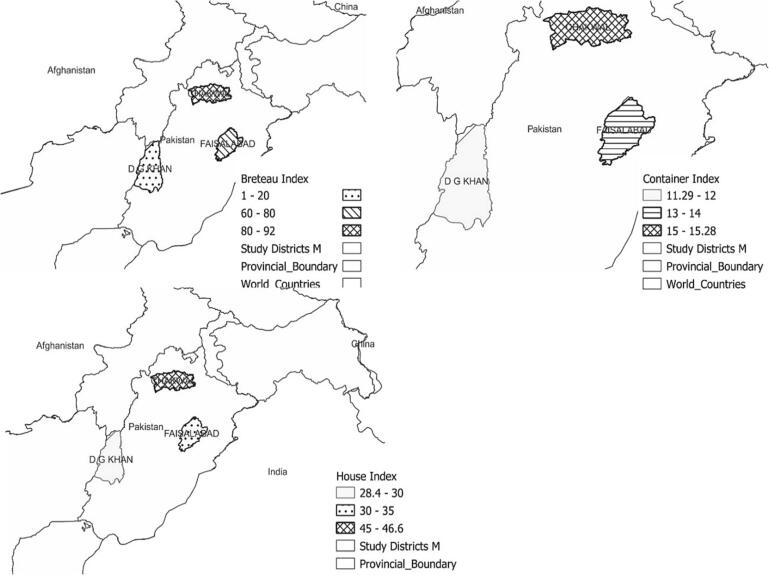
GIS mapping of stegomyia Indices in three representative districts of agrogeoclimatic zones of Punjab, Pakistan

## 4 Discussion

The *Stegomyia* indices HI, BI and CI in all the study districts were higher than the threshold level indicated by WHO. These findings are in accordance with the study done at Dire Dawa Eastern Ethiopia where these indices were recorded as 69.10 %, 134.55 % and 54 %, respectively (Getachew et al., 2015). On the contrary, the findings are different from another study conducted somewhere else (Dom et al., 2016) which found the larvae of *Ae. albopictus* breeding in domestic containers. The higher prevalence of *Ae. aegypti* in the present study may be attributed to the presence of appropriate water holding containers for breeding of *Ae. aegypti* and availability of organic material in the water for larval feeding which help maintaining the life cycle (Roy et al., 2021) and ultimately enhanced the risk of dengue virus transmission in the subjected population. The findings are also in line with a recent study published in India where a positive relation of breeding containers has been reported with the prevalence of *Ae*. *aegypti* in urban areas (Devi et al., 2023). However, contrary to the findings of the present study no significant association has been reported between the breeding containers and prevalence of *Ae. aegypti* in Bangladesh establishing multiple reasons e.g., obtaining data at single time interval and neglecting the outdoor breeding habitats which might aided in continuous presence of dengue larvae in the human population (Haque et al., 2023).

The association of plastic drums with the abundance of *Ae*. *aegypti* larvae throughout the year was found statistically insignificant (p > 0.05). The lower productivity of plastic containers has also been observed in West Indies, which attributed the production and survival of *Ae. aegypti* larvae in natural habitats to variation in the presence of micronutrients in water stored in natural habitats like tree holes as compared to artificial habitats like plastic containers (Hemme et al., 2020). On the contrary positive association of plastic drums have also been reported in Canada which stated that higher productivity of plastic containers may be attributed to the presence of excessive vegetation which ultimately mixed in the plastic containers thereby providing nutrients rich in nitrate and phosphorous responsible for growth and survivability of the larvae (Dom et al., 2016).

Insignificant association (p > 0.05) of flower tubs screened in DG Khan may be attributed to the reason that rural backgrounds or less developed areas have less interest in keeping plants indoor (Hemme et al., 2020). However, increased prevalence of *Ae. aegypti* larvae in developed areas i.e., Faisalabad, Chakwal may be attributed to the reason that lower numbers of larvae have been found from numerous flowerpots screened from the selected areas, making it negligible for people to recognize them as a source of mosquito larvae (Walker et al., 2018, Rahman et al., 2021).

Statistically significant (p < 0.05) association of plastic buckets in both districts Faisalabad and Chakwal, respectively may be attributed to the use of these buckets as source of water storage when water supply has been cut off from government (Colombani et al., 2016, Williams et al., 2019). Similar findings have been reported in Brazil, where significant association has been found between plastic buckets and abundance of *Ae. aegypti* larvae. The insignificant association of plastic buckets screened in DG Khan may be attributed to the water having higher concentrations of NaCl_2_, responsible for larval killing due to high salinity (Akhter et al., 2017).

Statistically insignificant (p > 0.05) association of refrigerator trays with the abundance of *Ae. aegypti* larvae in Faisalabad district is due to unavailability of refrigerator trays or lower numbers of refrigerators available in the screened population (Dhar-Chowdhury et al., 2016). However, significant association (p < 0.05) in DG Khan and Chakwal, respectively, which is in line with the findings of Elsinga et al. (2018) and Williams et al. (2019) attributed the higher productivity of refrigerator trays to human behavior and practice. Nowadays, affording refrigerators is very economical. Moreover, the provision of refrigerators is easy on the payment of that item in installments (Islam et al., 2019).

Significant statistical association (p < 0.05) of *Ae. aegypti* larvae and tyres in district Faisalabad and DG Khan may be attributed to the type of water retained in the tyres i.e., rainwater or pond water. The scientists reported that mosquitoes present in tyres having rainwater proliferate more actively as compared to pond water (Getachew et al., 2015, Ferreira-De-Lima and Lima-Camara, 2018). Insignificant association (p > 0.05) in district Chakwal may be attributed to the screening of tyres having water source other than rainwater, place of tyres either in shades or sunlight because *Ae. aegypti* preferred to breed in shady areas (Malla et al., 2020).

Insignificant association (p > 0.05) among the population of *Ae. aegypti* larvae and plastic bottles in district Faisalabad and Chakwal may be attributed to enough availability of water management resources and measures adopted to keep the water containers covered or dry after use (Aprisa et al., 2018, Ishak et al., 2020, Edillo et al., 2022). However, significant association (p < 0.05) at district DG Khan may be attributed to the domestic storage of tap and rainwater by the residents due to intermittent availability of drinking water supply and low rainfall. It has also been observed that people preferred rainwater for its use for laundry purpose rather tap water (Lin et al., 2018, Chumsri et al., 2018).

Water tanks screened from all the selected study districts were found statistically associated (p < 0.05). It has been reported that consistent population of the *Ae. aegypti* larvae was present in the supply lines or inside the sealed containers throughout winter season when the conditions were not favorable which shows that roof piping might have played the role in seeding water tanks with mosquito larvae during favorable conditions i.e., rainfall etc. (Jorge et al., 2019, Staunton et al., 2020). The role of gutters as breeding sites for *Ae. aegypti* larvae has also been reported previously. These gutters have high level of organic materials and stagnant water which is responsible for egg laying of mosquitoes and if somehow, while sealing the gutters, due to improper covering or sealing, small holes in seals, openings of mesh typical of water tanks having rainwater remained open would convert them to highly productive breeding habitats (Dieng et al., 2018, Chandrasiri et al., 2019).

The plastic sheets showed no significant association (p > 0.05) in any of the selected study districts. The factors responsible for lower numbers of larvae may include small surface area (Ross et al., 2019), small amount of water contained in the pits, exposed surface area to sunlight, daily cleaning of sheets by the residents, husk or hay stocks covered in a sloppy way to avoid retention of rainwater (Sinti-Hesse et al., 2019). On the contrary, positive association may be attributed to the larger surface areas surrounded by trees, retention of water, plastic sheets that were unattended by the residents, and improper cleaning (Britch et al., 2016).

Plastic bags that are thrown aimlessly, placed on the corners of the floor or roofs of the rooms or at the picnic places i.e., parks etc. were screened. The researchers attributed the significant association i.e., Faisalabad and Chakwal to social and behavioral practices of the people and level of hygienic measures adopted by the management authorities in the public places which remain unattended by the cleaning staff which served as potential source of breeding (Sultana et al., 2017). However, non-significant association i.e., DG Khan may be attributed to proper hygienic measures and continuous cleaning of public places (Cruvinel et al., 2020).

Non-significant association (p > 0.05) between *Ae. aegypti* larvae and plant axil in district Faisalabad and DG Khan may be attributed to the scarcity of rainfall leading to smaller volume of water retention in the plant axils (Brown et al., 2019), gravid females of *Ae. aegypti* mosquitoes also prefer to lay eggs in more than one batch (also called “skip oviposition”) and in more than one number of breeding places. However, significant association (p < 0.05) in district Chakwal may be attributed to the overlapping oviposition by multiple gravid females in same breeding container at one time (Suman et al., 2018, Gunathilaka et al., 2020).

Non-significant association (p > 0.05) between *Ae. aegypti* larvae and tree holes in district Faisalabad may be attributed to the presence less tree holes, more exposure to sunlight and small amount of water retention (Amarasinghe et al., 2019). However, significant association (p < 0.05) at both districts DG Khan and Chakwal may be attributed to the presence of higher number of trees (Walker et al., 2018), and the tree holes in vertical position having less exposure to sunlight as compared to horizontal position. The retention of rainwater for longer period has also been involved in aiding the larval habitat of the *Ae. aegypti* (Souza et al., 2019).

Earthen wares are also preferred breeding sites for the *Ae. aegypti* due to availability of shade, moisture, humidity, and ambient temperature. Significant association (p < 0.05) is observed in all the selected study districts. The researchers attributed the significant association of earthen jars to the characteristics of water present in the jar i.e., turbidity of water, alkalinity, pH and presence of macro and micronutrients in the water (Dom et al., 2016, Souza et al., 2019). The presence of vegetation and placement of jars in shady area have also been reported to be the probable reason for population of larvae. The non-significant association has also been reported from some researchers. The probable reason of non-significant association may include presence of heavy water (D_2_O), and low amount of oxygen present in the water (Kinamot et al., 2020).

Clay pots in district Faisalabad and DG Khan showed no statistical association (p > 0.05). The probable reasons of non-significant association may be attributed to the clay pots having direct sunlight exposure, water replacement by residents on daily basis, frequent use of clay pots by birds (Elsinga et al., 2018, Malla et al., 2020). However, significant association (p < 0.05) was observed in Chakwal whaich may be attributed to the clay pots that were kept in shady areas, unattended, and there was retention of rainwater in it (Souza et al., 2019, Gunathilaka et al., 2020).

## 5 Conclusion

The study was a maiden attempt to investigate the prevalence of *Ae. aegypti* immatures and their preferred breeding containers. The values of risk indices higher than threshold may be attributed to various factors i.e., the climatic conditions of the area like excessive rainfall or availability of natural or artificial source of water. Various biotic and abiotic factors might be overlooked while collecting the larvae or biasness in information on predesigned questionnaire and most importantly, water quality in the breeding containers which is not evaluated in the present study. Apart from these limitations, the study provided he baseline data regarding the presence of *Ae. aegypti* vector in the population of selected study areas. Therefore, the breeding containers identified should be subjected to appropriate control measures, such as source reduction via the removal of water-holding containers around living and working areas, and proper disposal of discarded materials should be implemented.

## Declaration of Competing Interest

The authors declare that they have no known competing financial interests or personal relationships that could have appeared to influence the work reported in this paper.
